# Preparation and Optimization of Fluorescent Thin Films of Rosamine-SiO_2_/TiO_2_ Composites for NO_2_ Sensing

**DOI:** 10.3390/ma10020124

**Published:** 2017-01-31

**Authors:** María G. Guillén, Francisco Gámez, Belén Suárez, Carla Queirós, Ana M. G. Silva, Ángel Barranco, Juan Ramón Sánchez-Valencia, José María Pedrosa, Tânia Lopes-Costa

**Affiliations:** 1 Departmento de Sistemas Físicos, Químicos y Naturales, Universidad Pablo de Olavide, Sevilla 41013, Spain; mariagonzalez88@gmail.com (M.G.G.); fgammar@gmail.com (F.G.); bsuarezjimenez@yahoo.es (B.S.); jmpedpoy@upo.es (J.M.P.); 2 REQUIMTE-LAQV, UCIBIO Departamento de Química e Bioquímica, Faculdade de Ciências da Universidade do Porto, R. Campo Alegre, Porto 4169-007, Portugal; up199800393@fc.up.pt (C.Q.); ana.silva@fc.up.pt (A.M.G.S.); 3 Instituto de Ciencia de Materiales de Sevilla, Universidad de Sevilla-CSIC, Américo Vespucio 49, Sevilla 41092, Spain; angel.barranco@csic.es (Á.B.); jrsanchez@csic.es (J.R.S.-V.)

**Keywords:** rosamine dyes, porous columnar semiconductors, gas sensors

## Abstract

The incorporation of a prototypical rosamine fluorescent dye from organic solutions into transparent and microstructured columnar TiO${}_{2}$ and SiO${}_{2}$ (MO${}_{2}$) thin films, prepared by evaporation at glancing angles (GAPVD), was evaluated. The aggregation of the adsorbed molecules, the infiltration efficiency and the adsorption kinetics were studied by means of UV-Vis absorption and fluorescence spectroscopies. Specifically, the infiltration equilibrium as well as the kinetic of adsorption of the emitting dye has been described by a Langmuir type adsorption isotherm and a pseudosecond order kinetic model, respectively. The anchoring mechanism of the rosamine to the MO${}_{2}$ matrix has been revealed by specular reflectance Fourier transform infrared spectroscopy and infiltration from aqueous solutions at different pH values. Finally, the sensing performance towards NO${}_{2}$ gas of optimized films has been assessed by following the changes of its fluorescence intensity revealing that the so-selected device exhibited improved sensing response compared to similar hybrid films reported in the literature.

## 1. Introduction

Xanthene dyes comprise a set of molecular architectures that possess particularly efficient absorptive and fluorescence properties. In combination with specific singularities acquired upon selective functionalization of the xanthene moiety, their applications have been progressively extended among a large landscape of technological areas [1,2,3,4,5,6]. For instance, the sensitizing effect provoked by the environment in rhodamine fluorophores has been widely employed as part of hybrid materials in fields as dye sensitized solar cells or chemosensing [7,8,9,10,11]. Moreover, their intense fluorescence emission allows their use as fluorescent probes, laser dyes, pigments, fluorescent standards or detection of reactive organic species [12,13,14,15]. These molecules are also widely used in biotechnology—for example, in the study of the membranes fluidity, the structure and dynamics of micelles, imaging in cells, and also in sensors for the detection of mercury, copper, iron and chromium in cells [16,17,18,19].

Although for some of these applications the dye is used in solution, most of them require this fluorescent probe to be anchored to a solid surface [20,21,22,23,24,25,26]. From a molecular viewpoint, establishing optimal values for the dye concentration, pH and solvent will determine the dominant chemical forms in the equilibrium, where cationic, zwitterionic, covalent dimers and *π*-*π* aggregates can exist. However, despite these cautions, when transferring to a solid matrix, the external potential represented by the solid walls in its pores makes the dyes even more prone to aggregation. This aggregation-inductive effect strongly challenges some practical goals by masking their solution-optimized emission properties when transferred to a rigid substrate. In this direction, materials science put at our disposal a wide plethora of functionalized substrates with predefined compositions and micro and nanostructures that present the adequate optical and electronic properties for very specific applications [27,28,29,30,31,32,33,34,35,36,37,38]. Remarkably, physical vapor deposition (PVD) methods allow us a very subtle and independent control of the structural and morphological parameters of the films, becoming an appropriate choice for the fabrication of hybrid materials [39]. Specifically, glancing angle physical vapor deposition (GAPVD) generates mainly columnar structures with micro and mesostructured porous surfaces [40,41,42,43] very suitable for the infiltration and incorporation of active molecules into photonic devices.

Additionally, since the core of these rhodamine dyes is constituted by a planar xanthene ring 3,6-disubstituted by in-plane amino groups and a nearby perpendicular 2${}^{\prime }$-carboxyphenyl ring in position 9, a pH-dependent behavior of the optical properties emerge as a consequence of the equilibrium between a fluorescent open ring and a non-fluorescent spirolactone forms [44]. Hence, in some circumstances, this feature goes hand-in-hand with the steric hindrance of the ring that preserves the anchoring to the substrate by the carboxylic substituent. Transposition of the carboxylic group from the 2${}^{\prime }$ to the 4${}^{\prime }$ positions suppresses these effects to a large extent. The resulting ensemble of molecules obtained within this positional change is denominated rosamine and its optimized synthetic procedure has been reported elsewhere [45]. The similarities between rhodamines and rosamines make them candidates for similar applications avoiding the mentioned chemical challenges.

**Figure 1:**
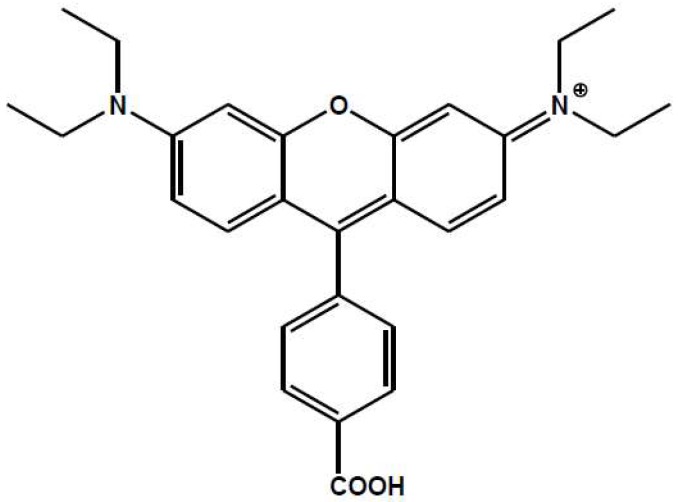
Schematic structure of the rosamine B molecular ion.

An innovative aspect of the present work is the coupling of both aspects by incorporating the rosamine shown in Figure 1 into non-dispersive TiO${}_{2}$ and SiO${}_{2}$ thin films prepared by GAPVD as hosting matrices. Taking into account the chemical structure of this rosamine, it can be anchored to the MO${}_{2}$ surface by electrostatic interaction between its ammonium group and the negatively charged surface of the oxide, or by chemical binding through its carboxylic group (well known in the field of dye sensitized solar cells). The spectroscopic properties of the films and the thermodynamics and kinetics of the dye incorporation process were evaluated. Their fluorescence response will be finally tested for the case of the best-behaved composite against the toxic NO${}_{2}$ gas which constitutes a very dangerous air pollutant for both environmental and human health [46].

## 2. Results and discussion

### 2.1. Spectroscopic Characterization of RosB/MO${}_{2}$ Composites: Molecular Aggregation

The RosB absorption spectrum in CH${}_{2}$Cl${}_{2}$ is constituted by a monomer band at 564 nm and a vibronic shoulder (traditionally called dimer band) at 527 nm (see Figure S1 in the Supplementary Material Section). The difference in the monomer-dimer absorbance peaks is consistent with their rhodamine fluorophore analogue [47]. Prior to further measurements, the use of CH${}_{2}$Cl${}_{2}$ is justified here because, besides presenting a positive solvatochromic effect that is not of interest for this work, it disfavors the aggregation with respect to aqueous solutions, mainly due to the smaller dielectric sandwiching effect and the absence of intermolecular hydrogen bonding in the former solvent. This fact is clearly shown in Figure S1 in the Supplementary Material section, where the absorbance spectra of a diluted solution of RosB in water and in organic CH${}_{2}$Cl${}_{2}$ solvents are compared.

The higher value of the aggregate/monomer ratio in the aqueous solution is apparent as expected. However, considering that the shape of the excitation spectra was always found to be very similar to that of their absorption spectra, this fact has been understood as the existence of a single type of molecules/aggregates with a spectrum characterized by two bands. These bands occur at shorter and longer wavelengths in relation with the maximum absorption of the monomer and are labeled as *H* and *J* bands, respectively [10]. An additional interpretation to this phenomenon can be mentioned: the aggregates can produce a similar emission to that of the monomers.

Regardless of the specific choice for the interpretation of the data, changes in the absorption profile can be unequivocally attributed to different ensembles of supramolecular arrangements. Hence, we will keep the classical first order exciton theory (ET) for the explanation of the experimental evidence. In this theory, these bands are attributed to the absorption of the dyes in dimeric forms with different mutual orientations labeled also as *H* and *J* (or Scheibe) dimers. These preformed aggregates play an important role in the emission properties of the supported composite. It is reported that dimers constitute efficient non radiative quenchers of the fluorescence by an internal conversion from charged-transfer states in their fundamental ground to excited singlet followed by an intersystem crossing to the charge-transfer triplet states [48]. In the way the preformed aggregates (mostly dimers) contribute to the total quenching upon deposition, it will be a factor to take into account in the choice of the optimum conditions for the highly emitting devices stated in the title. Although the Stokes shift (i.e., the difference between the maximum wavelengths of absorbance and fluorescence) clearly increases with RosB concentration (see Figure S2), no appreciable shape changes appear in the absorption spectrum of RosB in CH${}_{2}$Cl${}_{2}$ within the concentration range explored in this study. This fact ascribed to reabsorption phenomena has been described in dyes with overlapping emission/absorption spectra due to the absence of dipole moment parallel to the long axis [49]. In any case, the quenching of the fluorescence emission with the dye concentration shows a well-defined behavior that could be visualized by means of the ratio between the fluorescence intensity and the absorbance at the excitation wavelength (fluorescence efficiency) as shown in Figure S3. In view of the comments that we have given above, this diminution in solution cannot be associated to an enhancement of the aggregation but to reabsorption.

**Figure 2:**
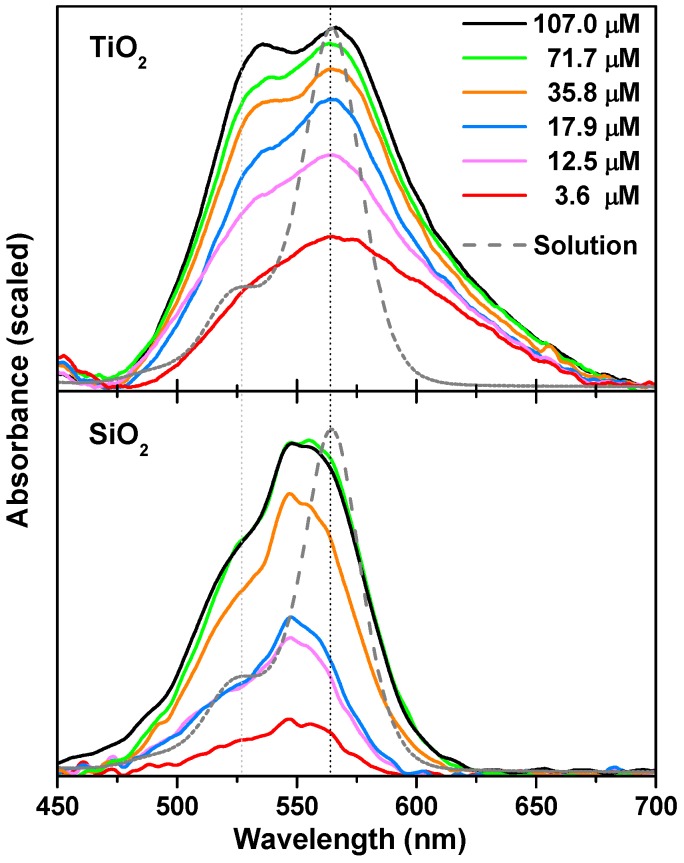
Absorption spectra for composite RosB/MO${}_{2}$ thin films prepared by infiltration of CH${}_{2}$Cl${}_{2}$ solutions at different concentrations. The infiltration time was 24 h in all cases. The corresponding spectrum in solution is plotted as dashed lines for the sake of comparison.

In Figure 2, the absorption spectra of the RosB infiltrated in MO${}_{2}$ films at an infiltration time of 24 h from different bulk concentrations is compared with the spectra of the CH${}_{2}$Cl${}_{2}$ solution at 3.5 μM for the sake of visual facilitation, considering that no appreciable aggregation contribution to the total amount of molecules in organic solution was observed. A general aspect of the adsorbed rosamine spectrum is that the absorption bands are broadened because of the interaction with the inorganic matrix and the formation of *H* aggregates as revealed by the progressive growth of the shoulder at ∼527 nm with the concentration of the RosB in the infiltrating solution. The stacking of RosB into non-covalent complexes is enhanced in TiO${}_{2}$ as observed in the comparison of both panels of Figure 2 as a consequence of the influence of the environment micropolarity and microacidity. This occurrence can be attributed to the higher acidic character of the surface hydroxyl groups in silanol with respect to titanol and to the charge compensation process that is the leading contributing effect to self-aggregation. Given the similarities with rhodamine B, rosamine B should own a pK${}_{a}$ value of ∼3 [50]. A zwitterionic form is then expected in this solvent allowing for a one- or two-sided anchoring to the surface charges of the matrices. Nevertheless, the point of zero charge (pzc) differs from ∼2.4 for SiO${}_{2}$ to ∼5.5 for TiO${}_{2}$ [43,51], and, hence, the charge balance between MO${}_{2}$ and the zwitterionic form of RosB would be differently equilibrated in the matrices, and the anchoring will also be unlike between them. This aspect will be discussed below by means of vibrational spectroscopy and infiltration of the matrices with aqueous solutions at different pH values.

In the limit of the diluted dye concentration, a broad distribution at longer wavelengths clearly emerges upon adsorption, which, according to ET, can be attributed to head-to-tail *J*-aggregates. The relative monomer/oligomer population decreases when the concentration increases (metachromatic effect) and also broadens and shifts to shorter wavelengths, which, provided *π* interactions with the free electrons of oxygen atoms in the surface are of minor importance in non-doped oxides [52], is indicative of an augmentation of the aggregation number of non-fluorescent sandwich-like *H*-aggregates.

Additionally, the blueshift of the monomer band in SiO${}_{2}$ (547 nm) is indicative of a more basic character of the environment created by the residual silanol groups and non-interacting amino groups within the pores of the amorphous silica columns with respect to the CH${}_{2}$Cl${}_{2}$ solution [53]. Remarkably, this band seems to have acquired a more structured shape with a new band at 555 nm. We tentatively assigned this feature to the existence of micro and mesoporous in the nanocolumns that will be detailed further along in this discussion. A plausible contribution to the splitting in the energy levels of the protonated and zwitterionic forms of the dye that are usually ill-resolved should also be mentioned [54]. *H*-dimerization is less accentuated, and a smaller aggregation number can be inferred from the nearly constant frequency of the dimer band. Therefore, a priori, rosamine infiltrated in SiO${}_{2}$ will provide better results in relation with its photoluminescence efficiency properties despite it throwing smaller absorbance intensity in comparison with TiO${}_{2}$ because the rosamine load is also smaller in these samples. These aspects are treated in detail in the following.

### 2.2. Spectroscopic Characterization of RosB/MO${}_{2}$ Composites: Adsorption Equilibrium and Kinetics

**Figure 3:**
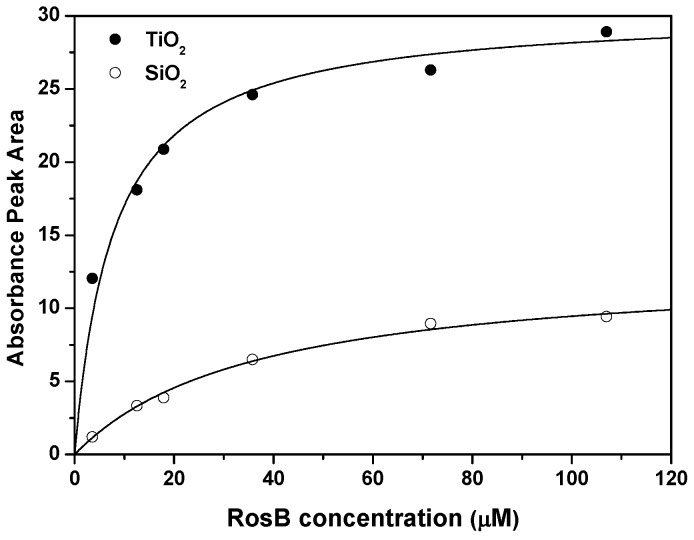
Adsorption behavior for RosB infiltrated in MO${}_{2}$ films. The corresponding fit to a Langmuir-type function is denoted with a continuous line while experimental data are labeled with circles.

Adsorption isotherms have been derived from the above results. The absorption band is integrated as a measure of the total amount of RosB molecules attached to MO${}_{2}$, i.e., the molecular surface concentration Γ [55]. The experiments were carried out by infiltrating RosB into the films during 24 h for a set of dye solutions with different concentrations as shown in Figure 3. The shape of the measured data indicates a high increment for small RosB concentrations to reach a high value at a solution concentration of around 40 μM and saturation is reached for more concentrated solutions. The so-obtained data are fitted to a Langmuir function of the form:

(1) $$A=\frac{C_{L}b\left[\mathrm{RosB}\right]}{1+b[\mathrm{RosB}]},$$

where *A* represents the integrated absorption band. The additional $C_{L}$ and *b* parameters stand for the adsorption capacity and affinity parameter in the Langmuir model. It should be mentioned that the fitting to Freundlich, Dubinin–Radushevich or Temkin models does not result in such an accurate reproduction of these points (R${}^{2}$ ≥ 0.99). The obtained parameters would put in front of the optimum performance for sensing to nanocolumnar TiO${}_{2}$ films, with a double number of adsorption sites and a higher affinity of the dye (in a factor 6 with respect to SiO${}_{2}$). However, most parts of the absorption band in TiO${}_{2}$ is due to non-fluorescent *H*-aggregates, and, hence, a notable quenching of the fluorescence properties will dominate these composites as we have commented on above.

The temporal behavior of the infiltration of the dye molecules into the columnar MO${}_{2}$ films has also been studied. The obtained experimental data is divided into a fast growth followed by a much slower process while approaching a saturation value $\Gamma_{\infty }$ (see Figure S4 in the Supplementary Material section). This pattern can be adjusted with the pseudosecond order adsorption kinetics model that has been tested for chemisorption of dyes into inorganic matrices [56], and it is supposed to account for the surface chemical bonds and physical diffusion steps. The model is the result of solving the following rate equation:

(2) $$\frac{d\Gamma (t)}{dt}=k_{2}{\left[\Gamma_{\infty }-\Gamma \left(t\right)\right]}^{2}$$

that can be straighforwardly integrated with the appropriate boundary condition (Γ(0) = 0) to get:

(3) $$\frac{1}{\Gamma_{\infty }-\Gamma \left(t\right)}=\frac{1}{\Gamma_{\infty }}+k_{2}t,$$

with $k_{2}$ the pseudosecond order adsorption rate. This equation can be linearized in several ways, and we have chosen [57]:

(4) $$\frac{t}{A(t)}=\frac{1}{k_{2}A_{\infty }^{2}}+\frac{1}{A_{\infty }}t$$

by considering again that the integrated absorption band *A* is proportional to Γ.

**Figure 4:**
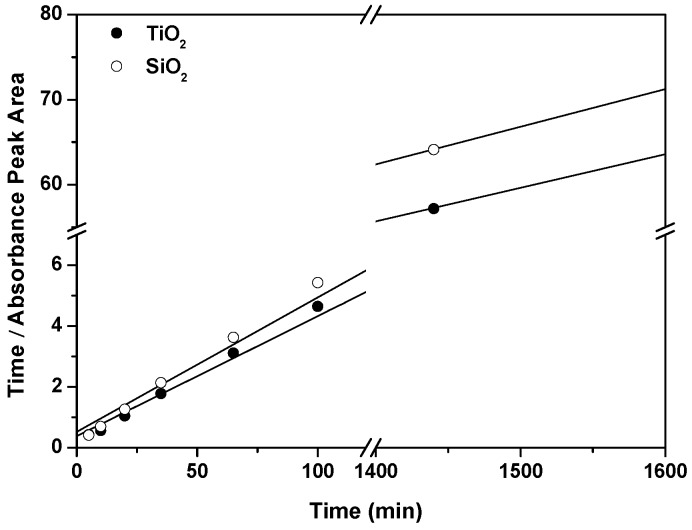
Linearized plot of the temporal evolution of the adsorption of RosB molecules infiltrated in MO${}_{2}$ films. The corresponding fit is denoted with a continuous line while experimental data are labeled with circles.

The result is plotted in Figure 4 for the 35.8 μM solution in the whole time range considered, with R${}^{2}$ ≥ 0.999. Similar fitting parameters are obtained for the two matrices, with ($A_{\infty }$, $k_{2}$) = (25.3, 0.0042) and (22.6, 0.0038) for TiO${}_{2}$ and SiO${}_{2}$, respectively, in units of [$A_{\infty }$] = nm and [$k_{2}$] = min${}^{-1}$. Together with the Langmuir fitting, it implies that these MO${}_{2}$ columnar microstructures exhibit a good infiltration capacity but also an excellent surface functionality for chemical adsorption and a good porous structure for the diffusive accessibility of the incoming rosamine molecules to the active adsorption sites.

### 2.3. Spectroscopic Characterization of RosB/MO${}_{2}$ Composites: Anchoring

**Figure 5:**
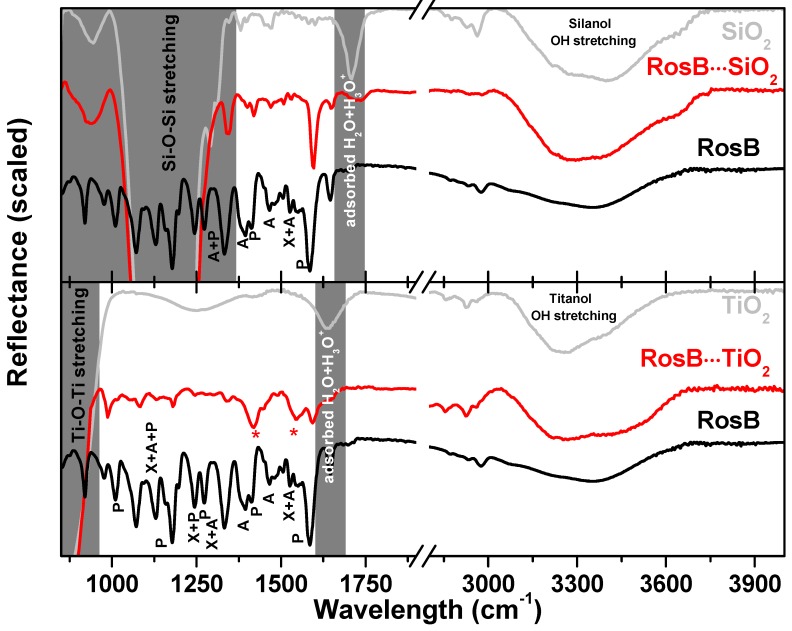
Specular reflectance Fourier Transform infrared spectra of MO${}_{2}$ and RosB precursors compared with the bounded RosB⋯MO${}_{2}$ in the devices. Vibration bands associated with phenyl ring (P). Amine (A) and xanthene ring (X) are indicated conveniently. See the main text for details.

As mentioned above, the molecular structure of RosB enables its anchoring to MO${}_{2}$ by chemical (through the carboxylic group) or electrostatical (through the amonium group) bounds. In Figure 5, the specular reflectance Fourier Transformed Infrared (FTIR) spectra of RosB and MO${}_{2}$ precursors have been compared with the bounded RosB in order to analyze the spectral changes in the region of the carbonyl and hydroxyl groups. Only the RosB bands with a major percentage (more than 50%) of potential energy distribution of normal modes of vibration located in the ethylamine (A), phenyl ring with the COOH group (P) or xanthene ring (X) groups are labeled according to the assignment reported in Ref. [58]. First of all, the strong absorption of the M-O-M asymmetric (transverse-optic and longitudinal-optic modes) stretching band clouds the signals coming from bounded RosB and hence this region is shadowed in the figure. In the 3300–3900 cm${}^{-1}$ region, there is the superposition of the hydrogen bond network of silanol and titanol groups and to the symmetric and antisymmetric OH modes of water coordinated to M${}^{4+}$ cations [59]. Finally the bending motion of the adsorbed H${}_{2}$O molecules appears at ∼1640 and ∼1710 cm${}^{-1}$ for TiO${}_{2}$ and SiO${}_{2}$, respectively [60,61]. The comparison of the RosB precursor with the bounded RosB spectra sheds light on to the mechanistic pathway of its surface anchoring. In the SiO${}_{2}$ substrate, the bands associated with the amine modes (A) disappear, at least partly, while the shape of the OH region remains unaltered with respect to the untreated substrate. This fact indicates an electrostatic anchoring of RosB to SiO${}_{2}$ and is in agreement with the absence of solid data supporting chemical bounds in carboxylic-dyes attached to silica matrices. On the contrary, in TiO${}_{2}$, both types of bands (A and P) suffer modification upon adsorption. Particularly, the bands at ∼1490, ∼1400 and ∼1180 cm${}^{-1}$ that are assigned to C=O, C-O and C-OH vibration become much weaker in favor of two new bands (marked with an asterisk) that correspond to the symmetric and asymmetric CO${}_{2}^{-}$ vibrations. These changes in the bands associated to P-vibrations have been reported to be compatible with chelating and/or bidentate binding modes of the carboxylate groups on the TiO${}_{2}$ surface. Overall, it seems plausible that RosB is anchored to TiO${}_{2}$ by two different ways: electrostatic or chemical bounds, which would explain the higher load derived for these films from Figure 3.

Complementary information can be extracted by infiltrating the dye from aqueous solutions at different pH values as reported in Figure S4 (Supplementary Information Section). The charge compensation on the inorganic matrix surface will depend on its pzc as explained above. In this sense, the immersion of a solution with a pH higher than its pzc results in a net negative charge on its surface by dissociation of the -OH (titanol or silanol) surface groups that is compensated by the cations in the double layer. With this point in mind and taking into account the pK${}_{a}$ value of the RosB molecule, in SiO${}_{2}$, the maximum load is reached for the measured pH which is closer to the pK${}_{a}$. If appreciable contribution of chemical anchoring takes place in these substrates, a much more evident increase near the point of charge compensation between RosB and MO${}_{2}$ would be seen. This is the case of TiO${}_{2}$ where the dye load is significantly enhanced near its pzc value and decreases with the pH because a deficient charge compensation of the excessive negative charge on the surface, i.e., the net negative charge in the surface is partly compensated by the positive charge of the zwitterionic RosB, but the overall charge remains negative, a fact that makes difficult a new approaching of another dye molecule. Thus, it is reconfirmed that while in TiO${}_{2}$ the anchoring occurs via chemical and electrostatic bonding, and this is not the case in SiO${}_{2}$ where an electrostatic mechanism seems to dominate the adsorption of RosB.

### 2.4. Photoluminescence of RosB/MO${}_{2}$ Composites: Choosing the Optimal Preparation Conditions for Enhancing the Fluorescence Signal

**Figure 6:**
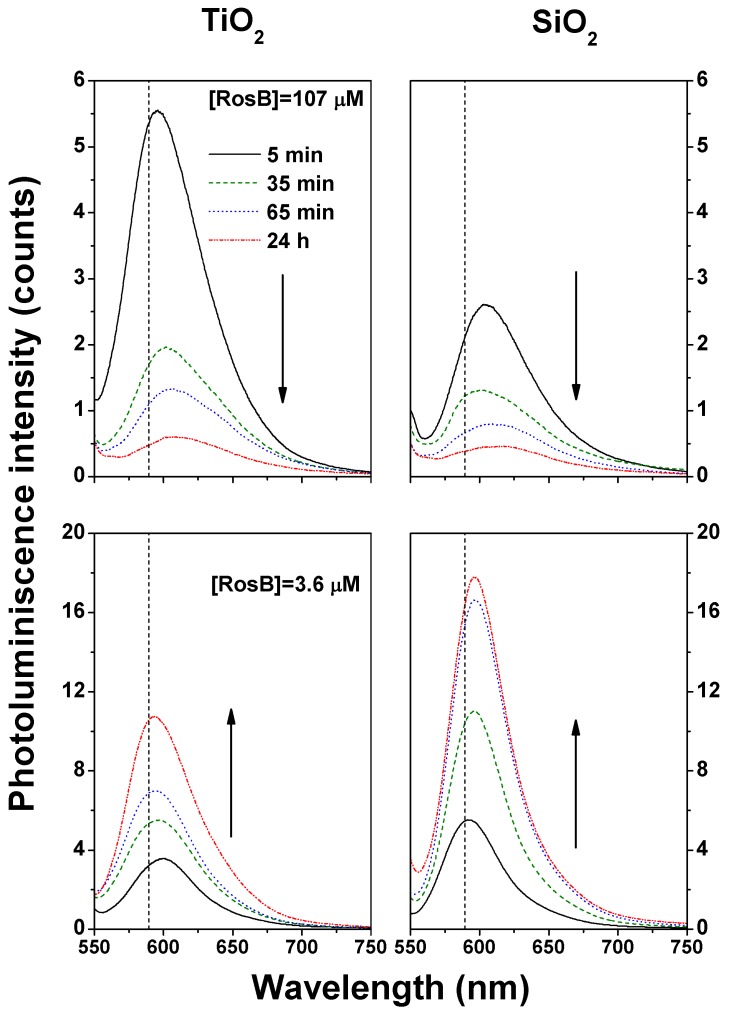
Photoluminescence spectra for composite RosB/MO${}_{2}$ thin films prepared by infiltration of dichloromethane solutions at the highest and lowest concentrations considered at different infiltration times.

As it has been discussed in the previous sections, whereas both monomers and aggregates are active in absorption, only the lowest excited state of dimers (the *J* dimers) and the monomers are fluorescent. Specific *J*-*H* conversion would require the measurement of fluorescence life times and is out of the scope of the present work. Fluorescence spectra constitute a mapping of (mainly) the monomeric state emission without the influence of the *H*-dimers and higher order aggregate quenching. Having this idea in mind, the interpretation of the temporal evolution of the infiltrated rosamine in MO${}_{2}$ presented in Figure 6 is eased. For the assistance of the subsequent explanation, the corresponding wavelength of the maximum fluorescence (589 nm) of the RosB monomer (3.5 μM) in CH${}_{2}$Cl${}_{2}$ solution is presented as a vertical line. This figure shows the temporal evolution of the RosB fluorescence infiltrated from two different concentration solutions in the two oxides. In both SiO${}_{2}$ and TiO${}_{2}$ films, the intensity evolution with infiltration time follows the same trend. The rosamine molecules infiltrated from a diluted solution increase their fluorescence intensity with time as a consequence of the smaller aggregation rate. If the concentration of the infiltrating solution is increased, the *H*-aggregation is apparent in the diminution of the intensity with the infiltration time in comparison with the diluted case, i.e., the emission is quickly quenched with infiltration time. This is also supported by the results shown in Figure 2. However, even when the fluorescence of SiO${}_{2}$ films is smaller than those obtained for TiO${}_{2}$ films, the first owns a comparably smaller loading capacity with respect to TiO${}_{2}$ as extracted with the corresponding Langmuir fitting. This point holds for both RosB concentrations with higher emission capacity for SiO${}_{2}$, again related to the lower rosamine aggregation observed in this material when compared to TiO${}_{2}$ (see Figure 2). Therefore, the films with the optimum optical properties are those constituted by a solid nanocolumnar SiO${}_{2}$ infiltrated with diluted RosB solution that exhibit the higher emission capacity in relation with the number of adsorbed molecules. It seems timely to mention that higher exposition times (≥24 h) lead to a diminution of the intensity even in the diluted cases because aggregation started to grow even in the open and wide mesoporous of their external surface.

The spectral details observed in the four cases can be explained by the opposing effects of aggregation and structural allocation of the dye. While *J*-aggregation induces a redshift of the photoluminescence bands, the fluorescence of the dye monomers adsorbed on different areas of the MO${}_{2}$ structure also provokes a shift evolution from monomers infiltrated to the inner parts or micropores of the oxide to a blue-shifted peak near to the solution maximum that corresponds to the outer-placed monomers. The same effect has been described in dyes adsorbed in different smectite (clay) minerals [62,63] in which the fluorescence of the adsorbed molecules at the external surface of smectites occurs at a lower wavelength than the corresponding fluorescence of Rhodamine 6G in the interlamellar space. This effect seems to be due to a geometrical constraint caused by the restricted space that reinforces the *π*-delocalization between both chromophores by a conformational-locking in which a more parallel disposition of the phenyl ring respect to the xanthene plane is favored [64]. Additional contributions of the microenvironment changes cannot be ruled out but do not explain the observed changes by themselves. In both MO${}_{2}$ films, the fluorescence intensity in the concentrated cases diminishes as a consequence of the *H*-aggregation, while *J*-aggregation induces a redshift with infiltration time as commented above. The structural effects of the inorganic matrix is negligible in these cases due to the high number of molecules filling the pores. In TiO${}_{2}$, we found a slight shift to the blue with infiltration time for the diluted RosB solution as a result of the adsorption from the inner (microporous) to the outer surface as it can be deduced from the closeness of the maximum fluorescence intensity with respect to the monomer emission in solution. The absence of a heterogeneous distribution of pores in SiO${}_{2}$ is the reason behind the redshift in the diluted case which can be attributed to *J*-aggregation as in the concentrated counterpart.

### 2.5. Sensing Response to NO${}_{2}$ Gas

**Figure 7:**
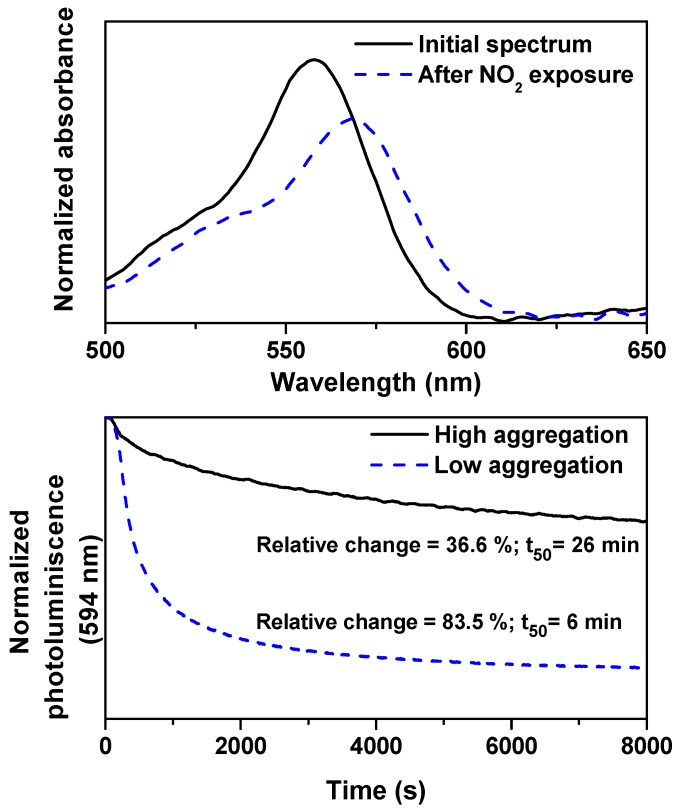
Top panel: spectral changes of the visible absorption spectrum of RosB (3.6 μM) bounded to SiO${}_{2}$ before and after NO${}_{2}$ (50 ppm) exposure. Bottom panel: photoluminescence response to NO${}_{2}$ (50 ppm) of RosB infiltrated in SiO${}_{2}$. The continuous line corresponds to 36 μM concentration (highly aggregated solution) and the dashed-lines stand for the 3.6 μM solution infiltration.

As a final application of the selected SiO${}_{2}$ microfilms infiltrated with diluted rosamine solution, we have tested the spectral changes provoked by an exposure to a NO${}_{2}$ stream (50 ppm). The observed spectral changes upon NO${}_{2}$ exposure documented in the top panel of Figure 7 (Δλ = 11 nm) demonstrate that rosamines bounded to a solid matrix constitute a good choice for gas detection. The most probable mechanism for such changes is possibly an oxidation reaction through a charge transfer process from the electron-rich xanthene ring to the oxidant gas [65,66,67]. Although the observed bathochromic shift is independent from the RosB concentration of the infiltrated solution, the intensity drop decreases with this concentration. For instance, the decrement of 23% observed for the most diluted solution is only 9% when the RosB concentration is quintupled (data not shown). This difference can be clearly explained in terms of the different aggregation state of the RosB molecules in these films: while low aggregation facilitates the analyte penetration and interaction, high molecular aggregation prevents this process [68]. The situation is even clearer when the more sensible fluorescence is tested as a sensing parameter, while, in the selected film with low aggregation state, the intensity falls off up to 84% and this drop is only of about 37% for a film infiltrated with a 36 μM solution.

The speed of the response is evaluated with the $t_{50}$ parameter, i.e., the time taken for the photoluminescence to reach 50% of its minimum value after NO${}_{2}$ exposure. The obtained value (6 min) is reasonably low and smaller than those obtained in recent porphyrin-based sensors (10.3 min) [69], thus confirming the fast response of SiO${}_{2}$. Conversely, the values of the $t_{50}$ increases up to 26 min for the aggregated case in SiO${}_{2}$ and is even higher in the TiO${}_{2}$ films with significantly reduced intensity changes. The higher aggregation in these latter samples precludes their use as gas sensors [68]. Additionally, only partial reversibility is reached after a recovery phase employing N${}_{2}$ while heating the samples. The concentration dependence of this response will be treated in a future work.

## 3. Conclusions

In the present work, the incorporation of a rosamine into transparent and microstructured columnar TiO${}_{2}$ and SiO${}_{2}$ thin films prepared by GAPVD have been investigated. The resulting composites exhibit low refractive indexes, high porosity, controlled thicknesses and robustness that, together with their spectroscopical properties, are judged as appropriate for photonic-based devices.

The concentration and time dependence of the infiltration process have been followed by spectroscopic measurements, and the results can be well described by a Langmuir and a pseudosecond order kinetics for the equilibrium and adsorption kinetic, respectively, discarding the physical diffusion to active adsorption sites as a limiting step in the dye infiltration. The spectroscopic features make evident that the aggregation rate is much more pronounced when the infiltration takes place from a concentrated solution and also in TiO${}_{2}$ films. The compensation of charges is different in the substrates because of their pzc, and, in consequence, the anchoring of the dye will also be different as demonstrated by FTIR. Therefore, a decrease in the fluorescence of the films with concentration and with infiltration time is the normal trend except for MO${}_{2}$ films infiltrated with a diluted RosB solution, in which the infiltration time also causes an increase of the fluorescence. Taking the absolute value of the emission intensity as the property of interest, the SiO${}_{2}$ film was tested as a NO${}_{2}$ sensor, indicating that the film response is fast and suitable for gas detection.

The strong influence of the interaction of the dye with the inorganic host has prompted us to perform experiments on different substrates that disfavor the *H*-aggregation into its surface and thus increase the sensoring response upon exposure to specific analytes [70]. Some preliminary advances on sinthered anatase TiO${}_{2}$ seem to be very promising and will be the topic of a future publication.

## 4. Materials and Methods

### 4.1. Chemicals

The synthesis of the xanthene dye [9-(4-carboxyphenyl)-6-diethylamino-3-xanthenylidene]- diethylamine chloride is fully described elsewhere [45]. Briefly, its synthesis involves a two-step sequence: (1) the microwave-assisted condensation of 3-(diethylamino)phenol with formylbenzoic acid in water and (2) oxidation with chloranil, followed by a chromatographic purification. The final molecular structure constitutes a positional isomer of rhodamine B, and, hence, it will be denoted as rosamine B (RosB) henceforth. Its optical properties in dichlorometane are also quite similar to those of rhodamine B [45], but the extinction coefficient at maximum absorption is only one-third of the rhodamine value. Dichlorometane was supplied by Sigma-Aldrich (Madrid, Spain) and was used as received.

### 4.2. Fabrication of MO${}_{2}$ Films and RosB Infiltration

Composite RosB/SiO${}_{2}$ and RosB/TiO${}_{2}$ thin films were prepared by using porous SiO${}_{2}$ and TiO${}_{2}$ thin films as inorganic host matrices. This matrices will be denoted as MO${}_{2}$ henceforth. The transparent (nondispersive) and amorphous TiO${}_{2}$ and SiO${}_{2}$ columnar films were prepared by the GAPVD technique at room temperature at an angle of deposition of 70${}^{\circ }$ with respect to the evaporation source. The microstructure of the films is evaluated by field emission scanning electron microscopy. Concretely, the angle formed by the columns and the substrate was approximately 60${}^{\circ }$, with a film thickness of approximately 350 nm. The mesostructure of the films is characterized by exhibiting a high porosity as determined by measuring the adsorption of water with a quartz crystal monitor corrected with the mass thickness of the thin film as determined by both Rutherford backscattering and X-ray fluorescence analysis (total pore volume of 49% in case of TiO${}_{2}$ films and 35% in case of SiO${}_{2}$ films). Therefore, coupled with electronic microscopies analysis, void apertures on the surface in the form of micropores (pore diameter ≤ 2 nm) in TiO${}_{2}$ and both micro and mesopores (pore diameter ≥ 2 nm) in SiO${}_{2}$ are observed. This feature would allow the accessibility of the rosamine during the composite film preparation and the NO${}_{2}$ molecules during the gas sensing experiments. The high porosity of the films also determines a refractive index value. By using absorption spectroscopy that was found to be relatively low (1.79) in the case of TiO${}_{2}$ films [71] and very low (1.317) in the case of SiO${}_{2}$ films [10], allowing their use in UV-Vis measurements. Further details regarding film preparation, SEM images and structural information can be found elsewhere [10,72]. For specular reflectance Fourier transform infrared (FTIR) spectroscopy, the films were deposited on gold-coated silicon substrates, whereas films for UV-Visible spectroscopy were prepared on glass substrates.

Binding of rosamine to the TiO${}_{2}$ and SiO${}_{2}$ films were performed by immersion of the substrates in several dichloromethane solutions of the dye. Afterwards, the films were rinsed with dichloromethane to remove dye molecules that were not incorporated into the film, and dried in air. Prior to the measurements, all films were cleaned with a dry N${}_{2}$ stream.

### 4.3. Instrumentation

UV-Visible absorption spectra were recorded using a Cary 100 UV-Vis spectrophotometer (Agilent, Madrid, Spain). Photoluminescence spectra and sensing kinetics were recorded with a Hitachi F-7000 Fluorescence Spectrophotometer (Hitachi High Technologies, Krefeld, Germany). For exposures to NO${}_{2}$, RosB/MO${}_{2}$ films were placed in a purpose-modified sealable fluorescence cuvette with a gas inlet and an outlet. Two Bronkhorst F-201FV mass flow controllers (Bronkhorst High-Tech BV, Ruurlo, The Netherlands) were used to control the flow rates of gases. Dry N${}_{2}$ was flowed into the cuvette before introducing the sample in order to remove any possible contaminating gases. After placing the sample in the cuvette, a constant dry N${}_{2}$ flow was kept to protect it during thermal stabilization. A constant flow of NO${}_{2}$ (50 ppm) was obtained from a cylinder from Abelló-Linde (Cádiz, Spain) Linde and introduced into the cuvette until complete saturation of the RosB. Samples were exposed at room temperature. Finally, dry N${}_{2}$ was made to flow into the cuvette during the recovery stage.

Specular reflectance FTIR spectroscopy experiments in RosB/MO${}_{2}$ films were performed using a Bruker IFS 66/S spectrometer (Bruker, Madrid, Spain). The background signal obtained from a gold substrate was subtracted in all cases. The infrared spectrum of rosamine B powder was measured in attenuated total reflectance (ATR) mode in a Jasco FT/IR-4100 espectometer (Jasco Inc., Easton, PA, USA) in an ATR Pro One module with an incidence angle of 45${}^{\circ }$. All spectra were obtained using typically 200 scans with a resolution of 4 cm${}^{-1}$.

## Supplementary Materials

The following are available online at www.mdpi.com/1996-1944/10/2/124/s1. Figure S1: Normalized absorption spectra of RosB in dichloromethane and water solutions at a 3.5 μM concentration, Figure S2: Concentration dependence of the photoluminescence spectra of RosB in dichloromethane. The absorption spectra of the most diluted solution is shown for the sake of comparison. The Stokes’s shift in the photoluminescence spectra with the dye concentration is due to reabsortion phenomonema as explain in the main text, Figure S3: Emission capacity of the RosB in dichlorometane solution as a function of the concentration. The quenching of fluorescence can be attributed to reabsortion phenomena, provided the negligible effect of the dye concentration in the absorption spectra, Figure S4: Temporal evolution of the adsorption of RosB molecules infiltrated in MO${}_{2}$ films, Figure S5: Evolution of the absorption band as a function of the pH during the infiltration procedure in MO${}_{2}$ substrates from aqueous solutions.
