# Supplementary material for: Preparation and Optimization of Fluorescent Thin Films of Rosamine-SiO2/TiO2 Composites for NO2 Sensing
Source: Materials (Basel). 2017 Jan 31;10(2):124. doi: 10.3390/ma10020124 (PMC5459166; doi:10.3390/ma10020124)
Supplement: Supplementary file 1 [file materials-10-00124-s001.pdf]

Supplementary Materials: Preparation and Optimization of Fluorescent Thin Films of Rosamine-SiO_2/TiO_2 Composites for NO_2 Sensing

María G. Guillén, Francisco Gámez, Belén Suárez, Carla Queirós, Ana M. G. Silva, Ángel Barranco, Juan Ramón Sánchez-Valencia, José María Pedrosa and Tânia Lopes-Costa

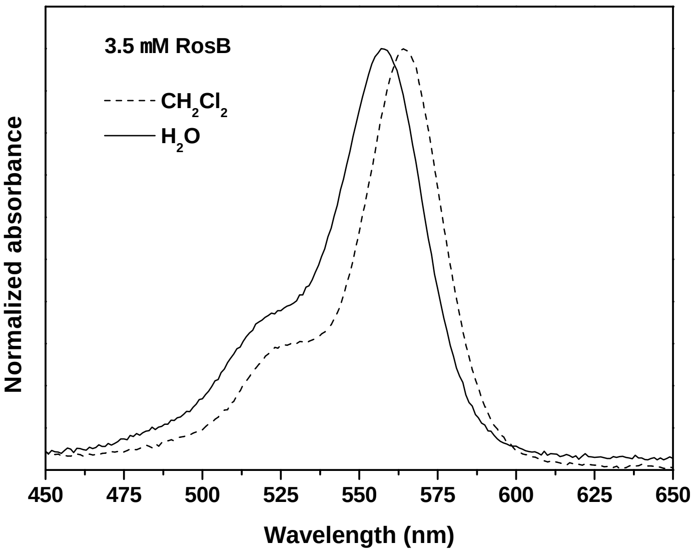

Figure S1. Normalized absorption spectra of RosB in dichloromethane and water solutions at a 3.5 µM concentration.

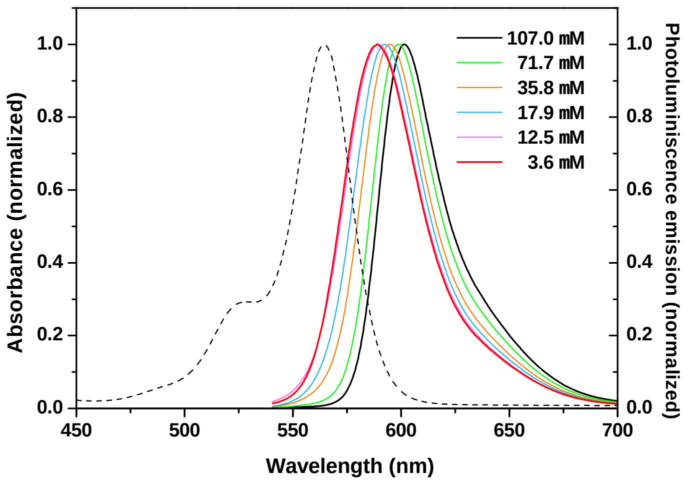

Figure S2. Concentration dependence of the photoluminescence spectra of RosB in dichloromethane. The absorption spectra of the most diluted solution is shown for the sake of comparison. The Stokes's shift in the photoluminescence spectra with the dye concentration is due to reabsortion phenomonema as explain in the main text.

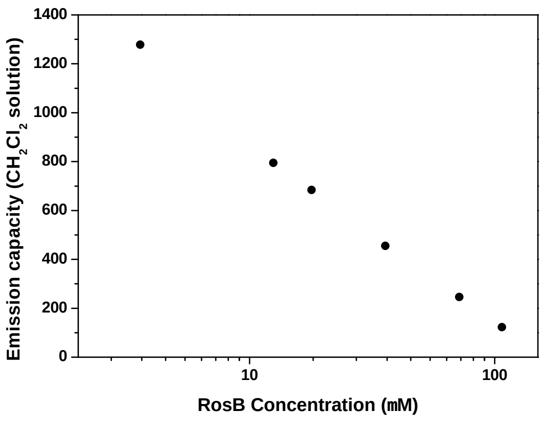

Figure S3. Emission capacity of the RosB in dichlorometane solution as a function of the concentration. The quenching of fluorescence can be attributed to reabsortion phenomena, provided the negligible effect of the dye concentration in the absorption spectra.

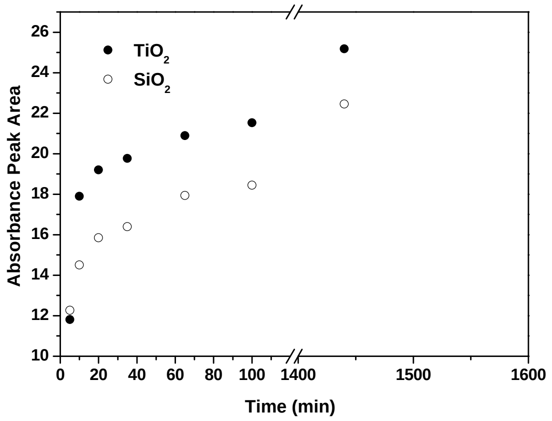

Figure S4. Temporal evolution of the adsorption of RosB molecules infiltrated in MO_2 films.

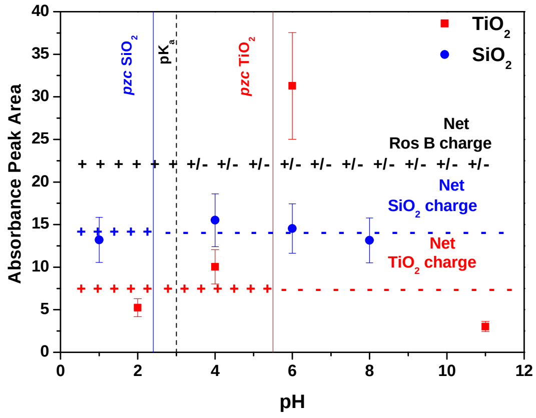

Figure S5. Evolution of the absorption band as a function of the pH during the infiltration procedure in MO_2 substrates from aqueous solutions. The *pzc* of each semiconductor oxyde and the pK_a value of the RosB dye are plotted as vertical lines. The net surface charge of MO_2 and the RosB charge as a function of the pH are correspondingly denoted with $+$ and $-$ symbols. See main text for details.
